# Immune network analysis of cerebrospinal fluid in myalgic encephalomyelitis/chronic fatigue syndrome with atypical and classical presentations

**DOI:** 10.1038/tp.2017.44

**Published:** 2017-04-04

**Authors:** M Hornig, C G Gottschalk, M L Eddy, X Che, J E Ukaigwe, D L Peterson, W I Lipkin

**Affiliations:** 1 Center for Infection and Immunity, Columbia University Mailman School of Public Health, New York, NY, USA; 2 Department of Epidemiology, Columbia University Mailman School of Public Health, New York, NY, USA; 3 Sierra Internal Medicine at Incline Village, Incline Village, NV, USA; 4 Department of Neurology, College of Physicians & Surgeons, Columbia University, New York, NY, USA; 5 Department of Pathology and Cell Biology, College of Physicians & Surgeons, Columbia University, New York, NY, USA

## Abstract

Myalgic encephalomyelitis/chronic fatigue syndrome (ME/CFS) is a persistent and debilitating disorder marked by cognitive and sensory dysfunction and unexplained physical fatigue. Classically, cases present after a prodrome consistent with infection; however, some cases are atypical and have a different presentation and comorbidities that pose challenges for differential diagnosis. We analyzed cerebrospinal fluid (CSF) from 32 cases with classical ME/CFS and 27 cases with atypical ME/CFS using a 51-plex cytokine assay. Atypical subjects differed in cytokine profiles from classical subjects. In logistic regression models incorporating immune molecules that were identified as potential predictor variables through feature selection, we found strong associations between the atypical ME/CFS phenotype and lower CSF levels of the inflammatory mediators, interleukin 17A and CXCL9. Network analysis revealed an absence of inverse inter-cytokine relationships in CSF from atypical patients, and more sparse positive intercorrelations, than classical subjects. Interleukin 1 receptor antagonist appeared to be a negative regulator in classical ME/CFS, with patterns suggestive of disturbances in interleukin 1 signaling and autoimmunity-type patterns of immune activation. Immune signatures in the central nervous system of ME/CFS patients with atypical features may be distinct from those with more typical clinical presentations.

## Introduction

Myalgic encephalomyelitis/chronic fatigue syndrome (ME/CFS) is a heterogeneous illness characterized by unexplained severe fatigue, sleep disturbance, pain and deficits in cognition and perception.^1^ Some investigators have reported evidence of immune activation or inflammation in the central nervous system (CNS) of subjects with ME/CFS. One study using ¹¹C-(R)-PK11195 PET—a ligand targeting a translocator protein that is expressed by activated microglia and astrocytes—described active inflammation in amygdala, thalamus and midbrain that correlated with patient-reported cognitive impairment.^2^ Other studies of the cerebrospinal fluid (CSF) proteome reported increased levels of proteins relating to the complement cascade.^3, 4^ In prior work defining CSF immune signatures in ME/CFS patients with classical presentations we found evidence of disrupted interleukin (IL)-1 signaling in CNS as compared with subjects with multiple sclerosis and no-disease controls.^5^ A proportion of patients who meet diagnostic criteria for ME/CFS at the onset of their illness have either a remote history of relatively uncommon exposures (viral encephalitis, illness after foreign travel or blood transfusion, Gulf War Illness) or develop comorbid immune-mediated^6, 7^ or neurological^8, 9^ disorders years later. Here we report discrete immunological profiles in the CSF of subjects with ME/CFS who vary in clinical presentation and comorbidity. Our findings may have utility for future work on the pathogenesis of this complex disorder and enable therapeutic interventions that target host response.

## Materials and methods

### Study population

The study population was comprised of 32 ME/CFS cases with classical features and presentations and 27 ME/CFS cases with atypical features or clinical presentations. CSF data for all 32 cases in the classical group were previously reported in an extended comparison with patients with multiple sclerosis (MS) and no-disease controls.^5^ All cases (both classical and atypical) met either the 1994 CDC criteria^10^ and/or the 2003 Canadian consensus criteria.^11^ The ‘classical' (C-ME/CFS) group had acute onset of disease marked by a prodrome consistent with infection; ‘atypical' (A-ME/CFS) ME/CFS patients met full diagnostic criteria for ME/CFS at onset of their illness, but had a less standard onset of ME/CFS and/or developed other disorders after illness onset of ME/CFS. Onset for the A-ME/CFS group was acute or non-acute with or without flu-like symptoms at the time of disease onset. This group tended to have more severe cognitive dysfunction and other neurological complaints. Some A-ME/CFS patients subsequently developed seizures and atypical MS or autoimmune/inflammatory disorders. Atypical MS and autoimmune/inflammatory disorders were categorized together in the ‘Immune or inflammatory' category of atypical ME/CFS. The ‘Other' category included patients with seizure disorder and the single subject with Gulf War Illness. Individuals who developed ME/CFS after blood transfusion or foreign travel were classified together in the ‘Probable infection' category. The last A-ME/CFS category comprised subjects who developed malignancies after their initial diagnosis of ME/CFS (Table 1). All subjects had contributed CSF samples to biobanks at Sierra Internal Medicine (SIM), a private internal medicine clinic in Incline Village, Nevada, where they were followed over subsequent years. Based on prior work on plasma cytokines in ME/CFS showing differences according to phase of the illness,^12^ we categorized duration of illness as either short (⩽3 years) or long (>3 years).

### Human subjects and ME/CFS biological sample collection

CSF collections followed standardized office protocol at SIM. Potential side effects or adverse events associated with specific clinically-indicated procedures such as lumbar puncture, as well as the clinical reason for these procedures, were discussed with patients in the course of their diagnostic work up and treatment planning. CSF was transferred by certified technicians from the lumbar puncture kit collection tubes into 2 ml Nalgene cryostorage tubes (Fisher Scientific, Pittsburgh, PA, USA). Excess CSF not consumed during indicated diagnostic tests was immediately stored in a −80 °C freezer located at SIM. Informed consent was obtained at the same time to allow any excess sample to be de-identified for use in any future research investigations of SIM.

### Selection of case and comparator samples

CSF samples were retrieved from SIM repositories and shipped on dry ice to the Wisconsin Viral Research Group (WVRG) where they were frozen in aliquots at −80 °C. CSF samples derived from the ME/CFS subjects had previously been clinically analyzed for oligoclonal banding, total protein, glucose and amino-acid profiles, helping to rule out other known contributory diagnoses such as MS. All CSF specimens submitted to WVRG represent archived diagnostic specimens exempt from HIPAA and institutional review board consideration (46.101 (b)(4), Code of Federal Regulations). All samples were de-identified prior to shipment to the Center for Infection and Immunity at Columbia University for analysis.

### Cytokine analyses

The CSF concentrations of the following immune molecules were determined using a magnetic bead-based 51-plex immunoassay: interleukin (IL)-1 superfamily, IL1α, IL1β, IL1RA; type I IL/γ chain family, IL2, IL4, IL7, IL13, IL15; type I IL/β chain family, IL5, GMCSF (CSF2); IL6 (gp130) family, IL6, LIF; IL12 family, IL12p40, IL12p70; IL10 family, IL10; IL17 family, IL17A, IL17F; type I interferons (IFN), IFNα2, IFNβ type II IFN, IFNγ tumor necrosis factor (TNF) superfamily, TNFα (TNFSF2), TNFβ (TNFSF1), CD40 ligand (CD40L), sFasL (TNFSF6), TRAIL (TNFSF10); CC chemokines, CCL2 (MCP1), CCL3 (MIP1α), CCL4 (MIP1β), CCL5 (RANTES), CCL7 (MCP3), CCL11 (eotaxin); CXC chemokines, CXCL1 (GROα), CXCL5 (ENA78), CXCL8 (IL8), CXCL9 (MIG), CXCL10 (IP10); PDGF family/VEGF subfamily, PDGFBB, VEGFA; cell adhesion molecules, sICAM1 (CD54), VCAM1 (CD106); serine protease inhibitor, serpin E1 (PAI1); adipose-derived hormones, leptin, resistin; and neurotrophic/growth/cellular factors, TGFα, TGFβ, FGFb, βNGF, HGF, SCF, MCSF (CSF1), GCSF (CSF3) (customized Procarta immunoassay, Affymetrix, Santa Clara, CA, USA). This cytokine panel was developed as an assay for investigating acute phase (‘sickness') responses and neuroimmune dysregulation in neuropsychiatric disorders that are postulated to be triggered by immune/infectious factors. It includes a wide range of cytokines, chemokines and cellular factors that reflect key processes relating to systemic activation of inflammatory/immune signaling pathways involved in autoimmunity and anti-inflammatory responses as well as others implicated in CNS inflammation and neurovascular disruption.

CSF samples were assayed in duplicate along with serial standards, buffer controls and in-house human control plasma samples.^13^ Samples from atypical and classical ME/CFS cases were run at the same time in randomized fashion on assay plates. Data from classical ME/CFS cases, with comparison to MS cases and no-disease controls, have previously been reported.^5^ Mean fluorescence intensities of analyte-specific immunoassay bead sets were detected by flow-based Luminex 3D suspension array system (Luminex, Austin, TX, USA).^14^ Cytokine concentrations were calculated by xPONENT build 4.0.846.0 (Luminex) and Milliplex Analyst software (v.3.5.5.0; VigeneTech, Boston, MA, USA) using a standard curve derived from the known reference concentrations supplied by the manufacturer. A five-parameter model was used to calculate final concentrations by interpolation. Values were expressed in pg ml^−1^. Concentrations obtained below the sensitivity limit of detection of the method were recoded to the mid-point between zero and the limit of detection for that analyte for statistical comparisons. Values obtained from the reading of samples that exceeded the upper limit of the sensitivity method were further diluted and re-assayed.

### Statistical analyses

Categorical demographic characteristics were compared by *χ*^2^-tests (sex, short vs long duration of illness) and continuous measures were compared by *t*-tests (age, duration of illness in years). Due to the overall small number of subjects in the A-ME/CFS cancer group, the heterogeneity of the types of cancer, and the highly variable time between CSF collection and development of the various types of neoplasia, these subjects were only included in a limited set of analyses (Supplementary Materials). The legend in Table 1 provides further detail on the composition of individual disorders or exposures within each of the atypical categories: (1) Cancer (*n*=8); (2) Immune or inflammatory (*n*=7); (3) Probable infection (*n*=5); (4) Other (*n*=7).

For each of the 51-plex cytokine assays, we compared the mean values in CSF cytokine levels between atypical ME/CFS cases (excluding subjects later-developing cancer) and classical ME/CFS cases, as well as between cases with shorter duration of illness (⩽3 years) and cases with longer duration of illness (>3 years), using two-sample *t*-tests. Each cytokine served as an individual hypothesis in this portion of the analysis, and thus we did not adjust for multiple comparisons. Because distributions deviated from normality, raw cytokine levels were first transformed using Box-Cox transformation defined as:


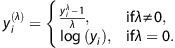


where for each cytokine variable, optimal λ was searched for to maximize the log-likelihood function.^15^ After being transformed, all variables failed to reject the null hypothesis using Kolmogorov-Smirnov test at the 0.05 level, confirming at the 95% confidence level that all transformed variables followed normal distributions. Analysis of variance were applied to examine both the main effects of atypical/classical ME/CFS status and duration of illness as well as their interaction effects, adjusting for various confounding variables including sex, age and storage time in years. As a *post hoc* analysis for the interaction effect between atypical/classical ME/CFS status and duration of illness, we created 4 subgroups within the study population, namely atypical short-duration cases, atypical long-duration cases, classical short-duration cases and classical long-duration cases, and for each cytokine, we compared mean values of its transformed levels between every pairwise combination of these 4 subgroups. For the 6 pairwise comparisons, we controlled the family-wise error rate at the 0.05 level using the Holm-Bonferroni correction procedure^16^ for multiple comparisons. A logistic regression model was built for prediction of the binary atypical/classical ME/CFS status variable using the data of the 51-plex cytokine assays. To eliminate multicollinearity among these predictor variables, two feature selection methods were used to guide selection of variables for the logistic regression model. Multiple methods were used to enable capture of factors potentially missed by one individual method. Raw cytokine levels were used in these analyses to allow for computation of odds ratios (ORs).

Feature selection methods select subsets of relevant features for use in regression model construction based on the assumption that the data contain many redundant or irrelevant features. We used two of the most widely used feature selection techniques, Lasso^17^ and Random Forests.^18^ Lasso (least absolute shrinkage and selection operator) regularizes the least squares by using the constraint that the L1 norm of the parameter vector is no greater than a given value, and increasing the penalty will cause more and more of the parameters to be driven to absolute zero. To avoid over-fitting, we selected the variables with non-zero coefficients when the cross-validation deviance reached the minimum plus one standard error.

The Random Forests method examines a large ensemble of decision trees by first generating a random sample of the original data with replacement (bootstrapping), and then using a number of variables selected at random from all of the variables to determine node splitting. The out-of-bag data is used to obtain a running unbiased estimate of the classification error as trees are added to the forest. Random Forests allows for calculation of measures of importance for each variable. Here we adopted two measures of importance: the mean decrease in accuracy due to the exclusion of the variable and the Gini index.^19^ The variables that were ranked in the top 5 in both measures were then selected for inclusion in the logistic regression model.

For the final logistic regression model with atypical/classical ME/CFS status as the dependent variable, a cytokine was entered if it was selected by Lasso or Random Forests. The ORs, their 95% confidence intervals (CIs), and *P*-values of the selected cytokines were calculated accordingly. Furthermore, we included the confounding variables including sex, age, duration of illness and storage time in years into the logistic regression model, and calculated the adjusted ORs together with their 95% CIs and *P*-values. In order to assess the goodness-of-fit of the final logistic regression models, we calculated area under the receiver operating characteristic curve (AUROC) and Bayesian Information Criterion values. Table 3 presents the ORs of all independent variables, their 95% CIs and *P*-values, and the goodness-of-fit information for both the unadjusted and adjusted logistic regression models.

The NodeXL platform was used to produce a network diagram of 51-plex assays within each group.^20^ The platform provides a display of the relationships among the analytes, thereby facilitating discovery of different cytokine–cytokine networks across the different group populations. Bivariate Pearson's correlations were first conducted between every pairwise combination of cytokine variables using their power-transformed (Box-Cox) values. We then used the Benjamini-Hochberg method to adjust for multiple comparisons with a 0.01 family-wise false discovery rate.^21^ Significantly correlated cytokine pairs were fed into the algorithm in NodeXL to produce the network diagram. Cytokines are represented by the ‘nodes' in the diagram, and significantly correlated cytokines are connected by ‘lines' or edges. Red lines represent negative correlations; gray lines represent positive correlations.

Statistical analyses were run in SPSS version 23.0.0.0, MATLAB version R2013a and R version 3.0.2.

## Results

Table 1 shows the demographic features of the overall study group comprising 32 C-ME/CFS subjects and 27A-ME/CFS subjects. A-ME/CFS subjects did not differ in age from the C-ME/CFS subjects (A-ME/CFS: mean±SD, 46.0±16.2 years vs C-ME/CFS: 49.9±11.2 years, *P*=0.283).

### A-ME/CFS cases vs C-ME/CFS cases

Excluding atypical cases with later-developing malignancies, we compared cytokine levels of A-ME/CFS subjects (*n*=19) with those of C-ME/CFS subjects (*n*=32) by 2-way analysis of variance (ME/CFS presentation type [atypical, classical] and illness duration [short, long]) adjusting for sex, age and sample storage time and found significant differences between atypical and classical ME/CFS cases in a large proportion of the 51 analytes (Supplementary Table S1). Significant atypicality × illness duration interactions were identified for IL1β (*P*=0.021); IL6 (*P*=0.027); IFNβ (*P*=0.034); TNFα (*P*=0.034); TNFβ (*P*=0.048); CCL2 (*P*=0.045); CSF3 (*P*=0.024) and βNGF (*P*=0.008).

**Figure 1:**
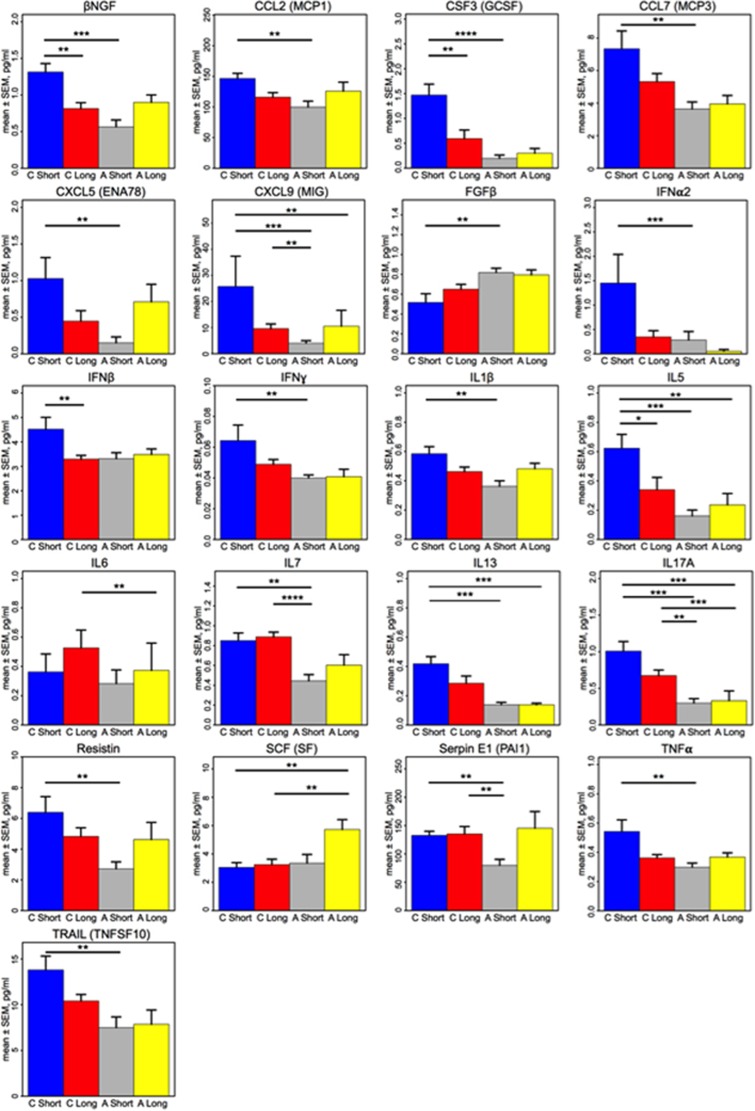
Comparison of levels of immune markers in cerebrospinal fluid (CSF) from classical myalgic encephalomyelitis/chronic fatigue syndrome (ME/CFS) and atypical ME/CFS cases. Comparison of cytokine levels (mean±s.e.m., in pg ml^−1^) in CSF from classical (C) and atypical (A) ME/CFS cases with short (⩽3 years) vs long (>3 years) illness duration. Only cytokines meeting significance criteria after Bonferroni–Holm correction for multiple comparisons are represented. *P<0.05, **P<0.01, ***P<0.001, ****P<0.0001 indicate *P*-values from two-sample *t*-test comparisons. IFN, interferon; IL, interleukin; TNF, tumor necrosis factor.

Post-hoc *t*-tests revealed prominent decreases in the levels of many pro- and anti-inflammatory cytokines in A-ME/CFS short-duration cases as compared with C-ME/CFS short-duration cases, including IL1β, IL5, IL7, IL13, IL17A, IFNα2, IFNγ, TNFα, TRAIL (TNFSF10), CCL2, CCL7, CXCL5, CXCL9, CSF3 (GCSF), βNGF, resistin and serpin E1 (PAI1). In contrast, only one analyte was increased in A-ME/CFS short-duration cases relative to C-ME/CFS short-duration cases: FGFb (*P*=0.005; Table 2; Figure 1).

Profiles of A-ME/CFS short-duration cases also differed from those of C-ME/CFS long-duration cases, with A-ME/CFS short-duration cases having lower levels of IL7 (*P*<0.0001), IL17 A (*P*=0.002), CXCL9 (*P*=0.004) and serpin E1 (PAI1; *P*=0.004).

When compared with C-ME/CFS short-duration cases, A-ME/CFS long-duration cases had significantly reduced levels of CSF IL5 (*P*=0.001), IL13 (*P*=0.0001), IL17 A (*P*=0.0002) and CXCL9 (MIG; *P*=0.007). In addition, A-ME/CFS long-duration subjects had lower levels of IL6 (*P*=0.001) and IL17 A (*P*=0.0002) than C-ME/CFS long-duration cases. In contrast, levels of SCF in CSF of A-ME/CFS long-duration subjects were higher than in C-ME/CFS subjects irrespective of duration of illness (C-ME/CFS short duration: *P*=0.001; C-ME/CFS long duration: *P*=0.002).

We next profiled CSF cytokines in the subset of A-ME/CFS subjects who developed malignancies after ME/CFS diagnosis (Supplementary Figure S1). A-ME/CFS subjects who developed malignancies (*n*=8) had higher levels of IL5, CSF2 and PDGFBB than other A-ME/CFS subjects (Supplementary Table S2). Routine CSF studies including glucose, protein and white blood cell counts revealed no major differences between A-ME/CFS subjects and C-ME/CFS subjects or the four C-ME/CFS exposure/comorbidity subgroups (Supplementary Table S3).

### Logistic regression models

After data reduction through feature selection procedures, variables meeting LASSO and Random Forests criteria (Supplementary Table S4) were included along with clinical covariates (age, sex, duration of illness and number of storage years) to construct the final logistic regression model and calculate the associated ORs, 95% CIs and *P*-values. Table 3 shows results for A-ME/CFS cases (excluding those with later malignancies) vs C-ME/CFS cases. In A-ME/CFS vs C-ME/CFS cases, lower levels of IL17A (OR, 0.00; 95% CI, 0.00, 0.85; *P*=0.047) were strongly associated with A-ME/CFS caseness as were lower levels of CXCL9 (MIG) (OR, 0.48; 95% CI, 0.25, 0.94; *P*=0.032).

### Network associations

**Figure 2:**
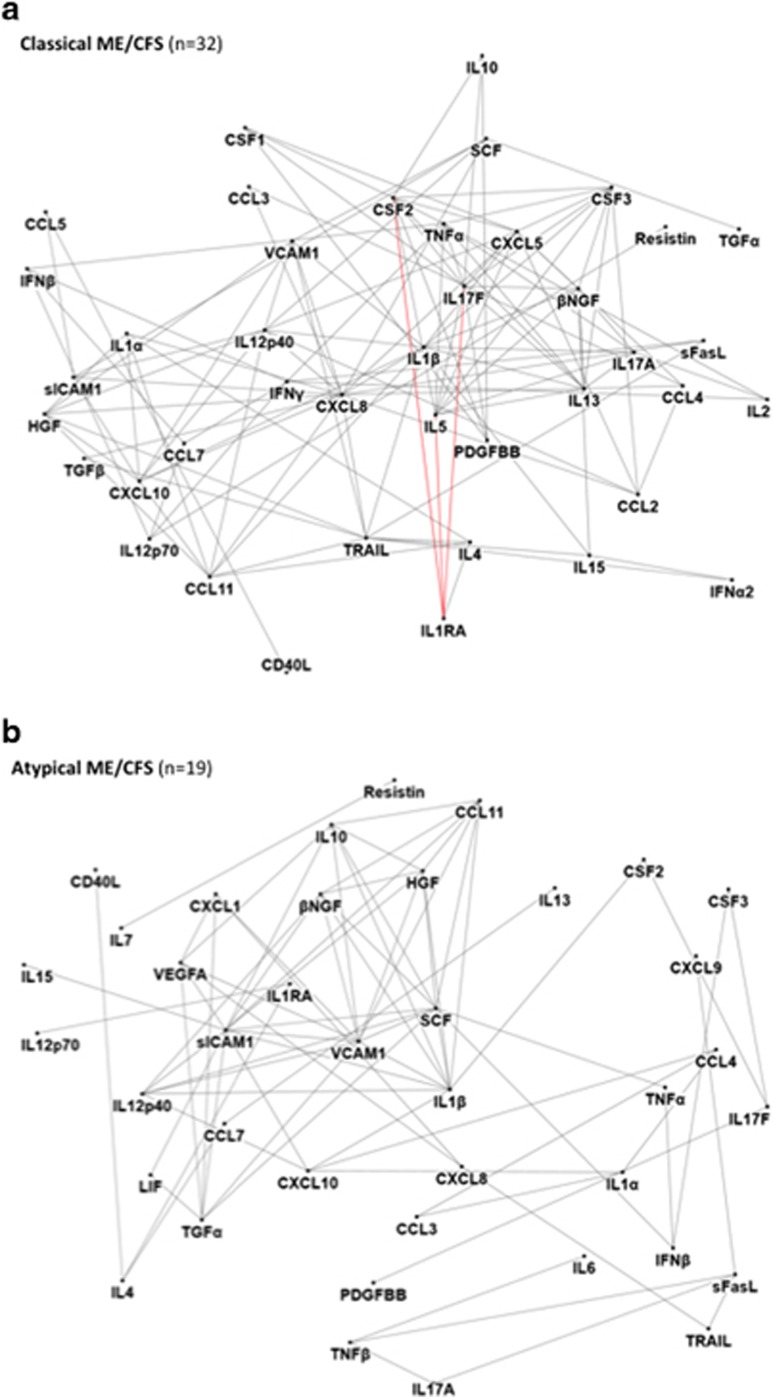
Network CSF cytokine–cytokine associations differ for classical and atypical ME/CFS cases. Network diagrams for classical myalgic encephalomyelitis/chronic fatigue syndrome (ME/CFS) subjects (**a**, *n*=32), and atypical ME/CFS subjects (**b**, *n*=19; excludes eight subjects later-developing malignancies). Network diagrams of the 51 measured cytokines were created in NodeXL (http://nodexl.codeplex.com) using a 0.01 family-wise false discovery rate (FDR) to adjust for multiple comparisons Red lines (edges) (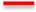) indicate negative correlations and gray lines (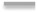) indicate positive cytokine–cytokine correlations with associated *P*-values that fall below the FDR-corrected *P*-value criterion for each group. For the classical ME/CFS group, IL1ra is inversely correlated with CSF2, IL5 and IL17F, and positively correlated with IL4. In contrast, in the atypical ME/CFS group, there are no inverse relationships among the very sparse set of cytokines represented. IL1ra is also correlated with IL4 in the atypical ME/CFS phenotype as in the classical ME/CFS group, but is also associated with the inflammatory cytokine, IL12p70. CSF, cerebrospinal fluid; IL, interleukin.

Network diagrams revealed unusual interrelationships among CSF cytokines in the A-ME/CFS group as compared to subjects with C-ME/CFS (Figure 2). C-ME/CFS subjects had inverse relationships between IL1ra and CSF2, and IL5 and IL17F, without correlation with IL1α or IL1β. In contrast, in A-ME/CFS subjects, IL1ra had only limited positive correlations, with IL4 and IL12p70. In addition, IL17A, which was an important predictor in our feature selection-driven logistic regression model, was richly interconnected with other cytokines in the C-ME/CFS CSF immune network, whereas in the A-ME/CFS group, IL17A was only correlated with TNFβ and sFasL.

## Discussion

Building on earlier work wherein we demonstrated distinct CSF immune signatures in subjects with a classical presentation of ME/CFS versus subjects with multiple sclerosis and no-disease controls,^5^ we compared CSF cytokine levels from the classical ME/CFS group with those from a group of ME/CFS cases who had atypical features at onset of illness or unusual comorbidities at the time of ME/CFS diagnosis. We found discrete differences in immune signatures of the CNS in ME/CFS subjects with atypical presentations that included sparse inter-cytokine networks and lower levels of two inflammatory mediators, the Th17 cytokine, IL17A, and the IFNγ- and TLR4-induced chemokine, CXCL9. Whereas network analyses showed that levels of IL1ra were inversely associated with CSF2, IL5 and IL17F in the classical ME/CFS group, there were no inverse associations among cytokines in the atypical group. These findings suggest potential differences in regulatory networks and less robust CNS immune activation in A-ME/CFS.

The strengths of this study lie in the quality of patient characterization; the large number of subjects with banked CSF samples, longitudinal surveillance for development of comorbidities and the use of a broad-based, sensitive immunoassay that captures components of the immune response relevant to neuroimmune signaling. Potential limitations, such as long storage times, were mitigated by minimization of freeze–thaw cycles and adjustments for length of storage time in the analysis. We excluded subjects in the cancer group from the main analysis due to concern that their physiology may have differed from other A-ME/CFS subjects and C-ME/CFS subjects prior to cancer diagnosis. The observation that our cohort included subjects with an A-ME/CFS profile who subsequently received a cancer diagnosis suggests that finding this profile should prompt a search for cancer, just as it does in paraneoplastic syndromes. Although few differences were apparent in cytokine levels across the different subsets within the A-ME/CFS subgroup, the atypical group as a whole had markedly lower levels of several inflammatory cytokines as compared with C-ME/CFS patients. Although some differences were noted in CSF cytokines based on duration of illness—a factor we previously found to profoundly affect plasma cytokine levels in ME/CFS in prior work^12^—adjustment for illness in our final logistic regression models did not eliminate findings of inhibited inflammatory cytokines in A-ME/CFS.

The importance of lower levels of IL17A and CXCL9 in the CNS in A-ME/CFS is unclear. We speculate that subjects with unusual exposures or comorbidities may have less activation of neuroimmune signaling pathways. In MS, CSF IL-17A levels are associated with disruption of the blood–brain barrier and expansion of neutrophils in CSF and directly correlated with CSF glutamate levels; IL-17A levels fall with disease duration, suggesting that glutamate toxicity may be more important in MS onset than in later stages.^22^ IL-17A levels tended to be lower in C-ME/CFS subjects with longer duration of illness; however, levels did not differ for atypical subjects based on disease duration. This may imply an alternate, non-Th17-dependent mechanism in atypical subjects, or a more rapid progression of neurodegeneration than in classical subjects. Recent evidence also suggests that IL-17A has minimal capacity to activate microglia.^23^ CXCL9 is also reported to be increased in CSF in relapsing MS, and decreases in conjunction with response to certain treatments, such as natalizumab.^24^

The deficits identified here in CNS interleukin 1 signaling among subjects with atypical presentations of ME/CFS, along with our finding of strong associations of very low levels of two inflammatory mediators with the atypical phenotype, suggest the potential value of vigorous pursuit of alternate, nonimmune mechanisms of pathogenesis in more complex, atypical patients with ME/CFS. Careful attention to exposure histories preceding onset of illness and longitudinal surveillance for the development of unusual medical comorbidities may help to identify novel pathways underlying dysfunction in this highly debilitated patient population.

## Figures and Tables

**Table 1:** Characteristics of the study population

*Variable*	*Atypical ME/CFS,* N=*27*	*Classical ME/CFS,* N=*32*	P*-value*
Sex, *n* (% female)	19 (70.4)	21 (65.6)	0.698^a^
Age (years), mean (s.d.)	46.0 (16.2)	49.9 (11.2)	0.283^b^
Duration of illness (years), mean (s.d.)	6.7 (7.8)	7.7 (6.9)	0.209^b^
			
*Duration of illness (categorical),* n *(%)*			*0.426*^a^
⩽3 years	15 (55.6)	14 (43.8)	
>3 years	12 (44.4)	12 (56.3)	
			
*Comorbidity or exposure type,* n *(%)*			
Cancer^c^	8 (29.6)	—	
Immune or inflammatory^d^	7 (25.9)	—	
Probable infection^e^	5 (18.5)	—	
Other^f^	7 (25.9)	—	

Abbreviations: ME/CFS, myalgic encephalomyelitis/chronic fatigue syndrome.

a Chi-squared test.

b Wilcoxon rank-sum test.

c Breast cancer (*n*=2); brain cancer (*n*=3); pancreatic cancer (*n*=1); leukemia/lymphoma (*n*=2).

d Atypical multiple sclerosis (*n*=3); autoimmune/inflammatory disorder (*n*=4).

e West Nile virus encephalitis (*n*=1); unspecified viral encephalitis (*n*=1); illness after foreign travel (*n*=2); illness after blood transfusion (*n*=1).

f Seizure disorder (*n*=6); Gulf War Illness (*n*=1).

**Table 2:** *T*-test comparison of CSF levels of cytokines and chemokines in atypical ME/CFS vs classical ME/CFS, by duration of illness^a^

*Immune molecule*	*Atypical short duration,* N=*13*	*Atypical long duration,* N=*6*	*Classical short duration,* N=*14*	*Classical long duration,* N=*18*	*Atypical short vs atypical long*	*Atypical short vs classical short*	*Atypical short vs classical long*	*Atypical long vs classical short*	*Atypical long vs classical long*	*Classical short vs classical long*
	*Mean (s.d.)*	*Mean (s.d.)*	*Mean (s.d.)*	*Mean (s.d.)*	P*-value*	P*-value*	P*-value*	P*-value*	P*-value*	P*-value*
IL1ra	9.80 (2.74)	11.01 (3.17)	10.65 (2.25)	11.26 (1.73)	0.404	0.398	0.086	0.759	0.840	0.405
IL1α	0.67 (0.48)	1.04 (0.50)	1.18 (1.38)	0.96 (0.86)	0.147	0.283	0.137	0.805	0.645	0.953
IL1β	0.36 (0.13)	0.50 (0.11)	0.58 (0.18)	0.46 (0.13)	0.040	**0.001**	0.047	0.347	0.482	0.038
IL2	0.02 (0.02)	0.02 (0.02)	0.07 (0.10)	0.03 (0.04)	0.999	0.100	0.073	0.215	0.160	0.755
IL4	14.56 (3.92)	16.36 (4.96)	16.81 (5.05)	16.63 (3.54)	0.399	0.187	0.139	0.839	0.910	0.849
IL5	0.15 (0.16)	0.12 (0.12)	0.62 (0.35)	0.34 (0.36)	0.660	**0.0002**	0.211	**0.001**	0.534	**0.012**
IL6	0.33 (0.37)	0.11 (0.22)	0.36 (0.46)	0.53 (0.51)	0.089	0.4986	0.096	0.010	**0.001**	0.245
IL7	0.48 (0.23)	0.65 (0.40)	0.85 (0.29)	0.89 (0.20)	0.282	**0.001**	**<0.0001**	0.206	0.199	0.657
CXCL8 (IL8)	4.00 (2.23)	3.69 (1.65)	5.40 (2.07)	4.56 (1.72)	0.873	0.067	0.270	0.068	0.214	0.217
IL10	0.34 (0.15)	0.46 (0.14)	0.49 (0.11)	0.43 (0.10)	0.135	0.010	0.087	0.662	0.518	0.116
IL12p40	0.11 (0.13)	0.28 (0.22)	0.20 (0.34)	0.09 (0.17)	0.122	0.816	0.330	0.177	0.015	0.217
IL12p70	0.40 (0.13)	0.40 (0.11)	0.59 (0.48)	0.45 (0.14)	0.911	0.095	0.272	0.185	0.345	0.351
IL13	0.14 (0.07)	0.15 (0.04)	0.42 (0.18)	0.29 (0.21)	0.530	**0.0002**	0.017	**0.0001**	0.017	0.057
IL15	0.75 (0.23)	0.94 (0.32)	0.95 (0.44)	0.77 (0.32)	0.141	0.157	0.859	0.992	0.254	0.196
IL17A	0.33 (0.23)	0.18 (0.23)	1.01 (0.49)	0.67 (0.32)	0.099	**0.0002**	**0.002**	**0.0002**	**0.0002**	0.055
IL17F	0.22 (0.06)	0.24 (0.06)	0.34 (0.28)	0.23 (0.07)	0.596	0.022	0.834	0.199	0.710	0.027
IFNα2	0.17 (0.51)	0.09 (0.16)	1.45 (2.20)	0.35 (0.54)	0.726	**0.0004**	0.042	0.011	0.230	0.049
IFNβ	3.24 (0.72)	3.62 (0.79)	4.52 (1.82)	3.29 (0.64)	0.326	0.012	0.776	0.236	0.362	**0.007**
IFNγ	0.04 (0.01)	0.04 (0.01)	0.06 (0.04)	0.05 (0.01)	0.581	**0.007**	0.050	0.035	0.082	0.111
TNFα	0.30 (0.11)	0.38 (0.11)	0.54 (0.30)	0.36 (0.10)	0.137	**0.006**	0.073	0.284	0.671	0.059
TNFβ	0.34 (0.16)	0.23 (0.17)	0.28 (0.15)	0.33 (0.12)	0.176	0.346	0.948	0.459	0.092	0.264
CD40L	0.88 (0.78)	0.92 (1.22)	1.85 (1.78)	1.28 (1.05)	0.686	0.075	0.216	0.113	0.194	0.379
sFasL	0.92 (0.47)	0.73 (0.49)	1.41 (0.97)	1.03 (0.22)	0.315	0.053	0.229	0.030	0.180	0.163
TRAIL (TNFSF10)	7.99 (4.40)	7.62 (4.05)	13.80 (5.65)	10.41 (3.01)	0.903	**0.007**	0.066	0.027	0.074	0.072
CCL2 (MCP1)	105.12 (27.98)	126.52 (50.59)	146.50 (31.63)	116.09 (31.08)	0.250	**0.001**	0.321	0.288	0.555	0.010
CCL3 (MIP1α)	2.99 (3.22)	2.99 (1.63)	4.01 (2.05)	4.34 (2.24)	0.620	0.134	0.067	0.355	0.151	0.611
CCL4 (MIP1β)	43.68 (50.90)	45.03 (28.10)	43.80 (27.75)	46.75 (27.09)	0.561	0.538	0.374	0.840	0.941	0.708
CCL5 (RANTES)	0.67 (0.69)	0.51 (0.44)	1.92 (3.28)	1.00 (1.87)	0.668	0.054	0.380	0.056	0.226	0.142
CCL7 (MCP3)	3.82 (1.73)	3.82 (1.99)	7.34 (4.11)	5.33 (2.08)	0.935	**0.003**	0.054	0.017	0.129	0.088
CCL11 (eotaxin)	1.85 (0.92)	2.93 (1.99)	3.46 (1.92)	2.61 (1.03)	0.127	0.012	0.032	0.579	0.734	0.212
CXCL1 (GROα)	3.02 (0.97)	3.01 (0.69)	3.53 (1.36)	3.41 (0.94)	0.824	0.234	0.212	0.400	0.388	0.909
CXCL5 (ENA78)	0.17 (0.34)	0.80 (1.05)	1.03 (1.07)	0.45 (0.60)	0.282	**0.002**	0.262	0.210	0.786	0.037
CXCL9 (MIG)	3.74 (3.20)	4.38 (4.65)	25.75 (43.34)	9.58 (7.62)	0.783	**0.0005**	**0.004**	**0.007**	0.114	0.038
CXCL10 (IP10)	37.30 (26.81)	59.76 (37.90)	79.31 (109.81)	55.45 (42.11)	0.240	0.148	0.084	0.915	0.907	0.781
TGFα	2.51 (1.02)	2.89 (0.83)	2.50 (0.64)	2.73 (0.76)	0.434	0.970	0.493	0.267	0.670	0.368
TGFβ	6.28 (6.92)	4.01 (3.72)	4.20 (4.39)	2.98 (2.68)	0.580	0.271	0.122	0.706	0.515	0.843
SCF (SF)	3.36 (2.28)	5.96 (1.65)	3.05 (1.27)	3.25 (1.61)	0.020	0.977	0.859	**0.001**	**0.002**	0.780
CSF1 (MCSF)	6.23 (4.16)	6.22 (6.94)	4.56 (3.00)	4.26 (2.91)	0.427	0.212	0.090	0.958	0.920	0.704
CSF2 (GMCSF)	0.43 (0.14)	0.54 (0.27)	0.73 (0.55)	0.54 (0.21)	0.422	0.014	0.161	0.303	0.863	0.182
CSF3 (GCSF)	0.18 (0.25)	0.37 (0.37)	1.47 (0.82)	0.59 (0.74)	0.442	**<0.0001**	0.356	0.040	0.994	**0.001**
PDGFBB	0.33 (0.13)	0.30 (0.09)	0.37 (0.38)	0.30 (0.12)	0.888	0.792	0.641	0.927	0.819	0.907
βNGF	0.59 (0.37)	0.85 (0.30)	1.31 (0.43)	0.82 (0.34)	0.111	**0.0001**	0.048	0.031	0.780	**0.001**
FGFb	0.83 (0.18)	0.80 (0.12)	0.52 (0.33)	0.65 (0.21)	0.733	**0.005**	0.016	0.011	0.102	0.172
HGF	28.88 (14.97)	35.53 (16.01)	32.01 (14.19)	27.69 (9.63)	0.360	0.546	0.937	0.614	0.170	0.371
VEGFA	5.60 (2.27)	6.30 (0.67)	6.74 (1.78)	6.37 (1.78)	0.280	0.143	0.269	0.460	0.938	0.559
LIF	0.22 (0.06)	0.28 (0.09)	0.31 (0.10)	0.25 (0.08)	0.091	0.036	0.326	0.691	0.457	0.165
Resistin	2.96 (1.72)	5.06 (3.78)	6.39 (3.84)	4.83 (2.37)	0.242	**0.003**	0.017	0.329	0.865	0.158
Leptin	44.07 (26.65)	66.35 (54.02)	55.44 (45.25)	78.13 (61.80)	0.433	0.678	0.143	0.683	0.728	0.307
Serpin E1 (PAI1)	83.74 (39.45)	123.36 (38.63)	132.69 (27.44)	135.25 (55.70)	0.053	**0.002**	**0.004**	0.489	0.679	0.943
sICAM1 (CD54)	96.44 (54.07)	150.05 (49.90)	135.97 (52.37)	135.96 (36.85)	0.061	0.048	0.030	0.560	0.496	0.937
VCAM1 (CD106)	342.23 (132.16)	431.50 (97.75)	375.29 (81.92)	336.56 (82.97)	0.145	0.367	0.974	0.199	0.031	0.185

Abbreviations: CSF, cerebrospinal fluid; IFN, interferon; IL, interleukin; ME/CFS, myalgic encephalomyelitis/chronic fatigue syndrome; TNF, tumor necrosis factor.

a Bold text indicates *P*-values meeting significance level after Bonferroni–Holm correction.

**Table 3:** Feature selection-driven logistic regression model for association of cerebrospinal fluid cytokines with atypical ME/CFS vs classical ME/CFS^a,b^

*Immune molecule*	*Unadjusted*				*Adjusted*^c^			
	*OR*	*95% CI*		P*-value*	*OR*	*95% CI*		P*-value*
IL7	**0.00**	**0.00**	**0.53**	**0.026**	0.01	0.00	76.66	0.316
IL17A	**0.01**	**0.00**	**0.51**	**0.023**	**0.00**	**0.00**	**0.85**	**0.047**
CXCL9 (MIG)	**0.75**	**0.56**	**1.00**	**0.050**	**0.48**	**0.25**	**0.94**	**0.032**
Model fit: AUROC	0.93				0.97			
Model fit: BIC	46.91				50.19			

Abbreviations: AUROC, area under the receiver operating characteristic; BIC, Bayesian Information Criterion, CI, confidence interval; IL, interleukin; ME/CFS, myalgic encephalomyelitis/chronic fatigue syndrome, OR, odds ratio.

a Feature selection via LASSO and Random Forests.

b 19 atypical ME/CFS subjects (excluding 8 atypical cases later-developing cancer); 32 classical ME/CFS subjects.

c Adjusted for sex, age, duration of illness and number of years of sample storage; bold text indicates *P*<0.05.
