# Supplementary material for: Immune network analysis of cerebrospinal fluid in myalgic encephalomyelitis/chronic fatigue syndrome with atypical and classical presentations
Source: Transl Psychiatry. 2017 Apr 4;7(4):e1080–. doi: 10.1038/tp.2017.44 (PMC5416687; doi:10.1038/tp.2017.44)
Supplement: Supplementary Information [file tp201744x1.doc]

**Supplementary Materials**

**Table S1. Two-way ANOVA comparing ME/CFS subjects with atypical and classical presentations and short vs. long duration of illness.^1,2^**

| **Immune**  **molecule** | **Diagnostic status** | | **Illness duration** | | **Diagnostic status x illness duration interaction** | |
| --- | --- | --- | --- | --- | --- | --- |
|  | Effect size**^3^** | *p*-value | Effect size**^3^** | *p*-value | Effect size**^3^** | *p*-value |
| IL1ra | 0.010 | 0.517 | 0.004 | 0.670 | 0.007 | 0.572 |
| IL1α | 0.003 | 0.726 | 0.002 | 0.773 | 0.009 | 0.542 |
| IL1β | 0.056 | 0.113 | 0.005 | 0.656 | **0.115** | **0.021** |
| IL2 | **0.131** | **0.014** | 0.006 | 0.597 | 0.010 | 0.499 |
| IL4 | 0.012 | 0.477 | 0.001 | 0.808 | 0.004 | 0.659 |
| IL5 | **0.147** | **0.009** | 0.035 | 0.212 | 0.045 | 0.157 |
| IL6 | **0.216** | **0.001** | 0.019 | 0.361 | **0.106** | **0.027** |
| IL7 | **0.191** | **0.002** | 0.002 | 0.770 | 0.006 | 0.614 |
| CXCL8 (IL8) | **0.111** | **0.024** | 0.033 | 0.224 | 0.000 | 0.983 |
| IL10 | 0.018 | 0.375 | 0.022 | 0.323 | 0.071 | 0.072 |
| IL12p40 | 0.078 | 0.060 | 0.002 | 0.788 | 0.042 | 0.174 |
| IL12p70 | **0.093** | **0.039** | 0.024 | 0.304 | 0.005 | 0.629 |
| IL13 | **0.227** | **0.001** | 0.022 | 0.326 | 0.050 | 0.134 |
| IL15 | 0.000 | 0.897 | 0.005 | 0.628 | 0.039 | 0.191 |
| IL17A | **0.473** | **0.000** | **0.100** | **0.032** | 0.008 | 0.543 |
| IL17F | 0.023 | 0.315 | 0.019 | 0.367 | 0.050 | 0.134 |
| IFNα2 | **0.255** | **0.000** | 0.003 | 0.724 | 0.046 | 0.151 |
| IFNβ | 0.018 | 0.377 | 0.050 | 0.135 | **0.099** | **0.034** |
| IFNγ | **0.206** | **0.002** | 0.055 | 0.118 | 0.011 | 0.491 |
| TNFα | 0.075 | 0.066 | 0.006 | 0.610 | **0.098** | **0.034** |
| TNFβ | 0.006 | 0.611 | 0.046 | 0.151 | **0.086** | **0.048** |
| CD40L | 0.051 | 0.133 | 0.080 | 0.056 | 0.001 | 0.842 |
| sFasL | **0.200** | **0.002** | 0.078 | 0.060 | 0.002 | 0.789 |
| TRAIL (TNFSF10) | **0.203** | **0.002** | 0.039 | 0.186 | 0.013 | 0.456 |
| CCL2 (MCP1) | 0.046 | 0.150 | 0.008 | 0.565 | **0.088** | **0.045** |
| CCL3 (MIP1α) | 0.074 | 0.066 | 0.005 | 0.625 | 0.000 | 0.952 |
| CCL4 (MIP1β) | 0.009 | 0.520 | 0.008 | 0.550 | 0.000 | 0.990 |
| CCL5 (RANTES) | **0.096** | **0.036** | 0.064 | 0.091 | 0.003 | 0.725 |
| CCL7 (MCP3) | **0.168** | **0.005** | 0.053 | 0.125 | 0.027 | 0.276 |
| CCL11 (eotaxin) | 0.033 | 0.224 | 0.019 | 0.361 | 0.039 | 0.191 |
| CXCL1 (GROα) | 0.034 | 0.221 | 0.055 | 0.116 | 0.001 | 0.851 |
| CXCL5 (ENA78) | 0.083 | 0.052 | 0.000 | 0.947 | 0.078 | 0.060 |
| CXCL9 (MIG) | **0.332** | **0.000** | 0.078 | 0.060 | 0.004 | 0.680 |
| CXCL10 (IP10) | 0.016 | 0.406 | 0.000 | 0.905 | 0.005 | 0.655 |
| TGFα | 0.019 | 0.364 | 0.001 | 0.868 | 0.000 | 0.912 |
| TGFβ | 0.007 | 0.581 | 0.003 | 0.721 | 0.013 | 0.454 |
| SCF (SF) | **0.165** | **0.005** | 0.039 | 0.186 | 0.065 | 0.087 |
| CSF1 (MCSF) | 0.013 | 0.450 | 0.032 | 0.234 | 0.012 | 0.460 |
| CSF2 (GMCSF) | 0.049 | 0.141 | 0.008 | 0.543 | 0.034 | 0.223 |
| CSF3 (GCSF) | **0.141** | **0.010** | 0.031 | 0.242 | **0.110** | **0.024** |
| PDGFBB | 0.012 | 0.478 | 0.007 | 0.592 | 0.001 | 0.813 |
| βNGF | **0.158** | **0.006** | 0.042 | 0.173 | **0.147** | **0.008** |
| FGFb | **0.198** | **0.002** | 0.000 | 0.976 | 0.031 | 0.240 |
| HGF | 0.024 | 0.307 | 0.028 | 0.264 | 0.007 | 0.591 |
| VEGFA | 0.019 | 0.367 | 0.002 | 0.767 | 0.011 | 0.489 |
| LIF | 0.008 | 0.554 | 0.003 | 0.712 | 0.043 | 0.166 |
| Resistin | 0.084 | 0.051 | 0.010 | 0.519 | 0.079 | 0.058 |
| Leptin | 0.002 | 0.748 | 0.006 | 0.611 | 0.002 | 0.771 |
| Serpin E1 (PAI1) | **0.152** | **0.007** | 0.001 | 0.833 | 0.023 | 0.317 |
| sICAM1 (CD54) | 0.005 | 0.635 | 0.008 | 0.547 | 0.047 | 0.148 |
| VCAM1 (CD106) | 0.030 | 0.253 | 0.002 | 0.755 | 0.053 | 0.125 |

**Key: ME/CFS**, myalgic encephalomyelitis/chronic fatigue syndrome

^1^ Atypical ME/CFS, n=19 (excludes 8 subjects later developing cancer); Classical ME/CFS, n=32; Short illness duration (≤3 years), n=27; Long illness duration (>3 years), n=24

^2^ Adjusted for sex, age and sample storage time in years; **bold text** indicates *p*<0.05

3 *Partial eta squared effect size*: as a general rule of thumb, small, medium and large partial eta squared effect sizes may be defined as 0.01, 0.06 and 0.14, respectively (Cohen, 1988). Here, all the large effects (>0.14) were identified as significant (*p*<0.05).

**Table S2. T-tests for comparison of levels of immune molecules in CSF of atypical ME/CFS cases with and without later development of cancer.**

| **Immune molecule** | **Atypical ME/CFS**  **Cancer group**^1^  N=8 | **Atypical ME/CFS**  **Non-cancer group**  N=19 | **Direction of effect** | ***p*-**  **value** |
| --- | --- | --- | --- | --- |
|  | mean (SD) | mean (SD) |  |  |
| IL1ra | 9.10 (4.84) | 10.18 (2.85) | ↓ | 0.525 |
| IL1α | 1.38 (1.08) | 0.79 (0.51) | ↑ | 0.069 |
| IL1β | 0.43 (0.18) | 0.41 (0.14) | ↑ | 0.764 |
| IL2 | 0.03 (0.04) | 0.02 (0.02) | ↑ | 0.842 |
| IL4 | 12.20 (5.05) | 15.13 (4.22) | ↓ | 0.147 |
| IL5 | **0.33 (0.29)** | **0.14 (0.15)** | **↑** | **0.041** |
| IL6 | 0.48 (0.77) | 0.26 (0.34) | ↑ | 0.905 |
| IL7 | 0.47 (0.36) | 0.54 (0.29) | ↓ | 0.575 |
| CXCL8 (IL8) | 4.84 (4.51) | 3.90 (2.02) | ↑ | 0.932 |
| IL10 | 0.40 (0.20) | 0.38 (0.16) | ↑ | 0.816 |
| IL12p40 | 0.27 (0.25) | 0.16 (0.18) | ↑ | 0.372 |
| IL12p70 | 0.42 (0.25) | 0.40 (0.12) | ↑ | 0.693 |
| IL13 | 0.13 (0.03) | 0.14 (0.06) | ↓ | 0.840 |
| IL15 | 0.64 (0.20) | 0.81 (0.27) | ↓ | 0.127 |
| IL17A | 0.38 (0.54) | 0.28 (0.24) | ↑ | 0.827 |
| IL17F | 0.27 (0.04) | 0.22 (0.06) | ↑ | 0.074 |
| IFNα2 | 0.27 (0.72) | 0.14 (0.42) | ↑ | 0.997 |
| IFNβ | 3.46 (1.18) | 3.36 (0.74) | ↑ | 0.875 |
| IFNγ | 0.04 (0.02) | 0.04 (0.01) | ↑ | 0.947 |
| TNFα | 0.34 (0.09) | 0.32 (0.12) | ↑ | 0.538 |
| TNFβ | 0.33 (0.24) | 0.30 (0.17) | ↑ | 0.808 |
| CD40L | 1.33 (1.65) | 0.89 (0.91) | ↑ | 0.962 |
| sFasL | 0.87 (0.61) | 0.86 (0.47) | ↑ | 0.962 |
| TRAIL (TNFSF10) | 7.15 (6.48) | 7.87 (4.18) | ↓ | 0.603 |
| CCL2 (MCP1) | 109.96 (64.73) | 111.88 (36.57) | ↓ | 0.922 |
| CCL3 (MIP1α) | 3.11 (4.87) | 2.99 (2.77) | ↑ | 0.641 |
| CCL4 (MIP1β) | 47.24 (83.94) | 44.11 (44.12) | ↑ | 0.385 |
| CCL5 (RANTES) | 0.67 (0.66) | 0.62 (0.62) | ↑ | 0.821 |
| CCL7 (MCP3) | 3.68 (1.67) | 3.82 (1.76) | ↓ | 0.973 |
| CCL11 (eotaxin) | 2.53 (1.84) | 2.19 (1.39) | ↑ | 0.853 |
| CXCL1 (GROα) | 3.51 (2.31) | 3.01 (0.87) | ↑ | 0.984 |
| CXCL5 (ENA78) | 0.47 (0.59) | 0.37 (0.69) | ↑ | 0.513 |
| CXCL9 (MIG) | 13.93 (25.62) | 3.94 (3.60) | ↑ | 0.985 |
| CXCL10 (IP10) | 91.25 (91.14) | 44.39 (31.51) | ↑ | 0.051 |
| TGFα | 2.55 (1.70) | 2.63 (0.96) | ↓ | 0.924 |
| TGFβ | 4.18 (5.35) | 5.56 (6.08) | ↓ | 0.460 |
| SCF (SF) | 4.93 (3.28) | 4.18 (2.40) | ↑ | 0.801 |
| CSF1 (MCSF) | 6.37 (4.47) | 6.23 (4.99) | ↑ | 0.921 |
| CSF2 (GMCSF) | **0.62 (0.15)** | **0.46 (0.19)** | **↑** | **0.029** |
| CSF3 (GCSF) | 0.25 (0.31) | 0.24 (0.30) | ↑ | 0.726 |
| PDGFBB | **0.41 (0.08)** | **0.32 (0.12)** | **↑** | **0.042** |
| bNGF | 0.81 (0.46) | 0.67 (0.36) | ↑ | 0.561 |
| FGFb | 0.78 (0.19) | 0.82 (0.16) | ↓ | 0.515 |
| HGF | 32.26 (17.87) | 30.98 (15.19) | ↑ | 0.977 |
| VEGFA | 6.58 (4.74) | 5.82 (1.92) | ↑ | 0.778 |
| LIF | 0.84 (1.59) | 0.24 (0.07) | ↑ | 0.454 |
| Resistin | 3.43 (3.90) | 3.62 (2.64) | ↓ | 0.645 |
| Leptin | 55.76 (41.98) | 51.10 (37.38) | ↑ | 0.901 |
| Serpin E1 (PAI1) | 139.08 (133.23) | 96.25 (42.54) | ↑ | 0.668 |
| sICAM1 (CD54) | 157.81 (102.54) | 113.37 (57.41) | ↑ | 0.233 |
| VCAM1 (CD106) | 371.25 (162.25) | 370.42 (126.95) | ↑ | 0.944 |

**Key:** **ME/CFS**, myalgic encephalomyelitis/chronic fatigue syndrome; **SD**, standard deviation

| Abnormality or unusual feature on  routine CSF analysis* | Atypical ME/CFS  N=18 subjects with  available data  (of total of 27 ) | Classical ME/CFS  N=28 subjects with  available data  (of total of 32) |
| --- | --- | --- |
| Total protein:  high | 1  (1 atypical MS) | 6 |
| Albumin:  low | 3  (1 cancer, 1 probable infection,  1 seizure disorder) | 2 |
| Albumin:  high | 0 | 1 |
| Glucose:  high | 1  (1 probable infection) | 1 |
| Lactic acid:  low | 1  (1 probable infection) | 5 |
| Oligoclonal bands:  1-3/equivocal (<4 considered WNL) | 1  (1 atypical MS) | 4 |
| Myelin basic protein:  high | 1  (1 cancer) | 1 |
| CSF polymorphonuclear cells:  high | 1  (1 cancer) | 0 |
| CSF lymphocytes:  high | 0 | 2 |
| CSF lymphocytes:  low | 1  (1 cancer) | 0 |
| CSF IgG/albumin ratio:  high | 1  (1 cancer) | 0 |
| CSF IgG index:  high | 1  (1 cancer) | 1 |
| CSF/serum albumin index:  high | 0 | 1 |
| Serum IgG, quantitative:  low | 0 | 1 |
| Anti-HSV type 1 or 2 antibody, screening IgG (CSF):  positive or equivocal | 3  (2 cancer, 1 autoimmune/  inflammatory) | 8  (5 positive;  3 equivocal) |
| Anti-HHV6 antibody, IgG (CSF):  positive | 0 | 1 |
| Anti-VZ virus antibody, complement fixation test (CSF):  positive | 0 | 1 |

**Table S3. Abnormalities detected on routine CSF assessment for study population.**

**Key: CSF**, cerebrospinal fluid; HSV, herpes simplex virus; Ig, immunoglobulin; ME/CFS, myalgic encephalomyelitis/chronic fatigue syndrome; MS, multiple sclerosis; VZ, varicella-zoster virus; WNL, within normal limits

* No CSF analysis abnormalities observed for the following parameters: % bands, monocytes, or eosinophils; anti-HSV type 1 or 2 antibody, IgM; anti-measles antibody, IgM or IgG; anti-mumps antibody, IgM or IgG; anti-Eastern equine encephalitis virus antibody, IgM or IgG; anti-Western equine encephalitis virus antibody, IgM or IgG; anti-California encephalitis virus antibody, IgM or IgG; anti-St. Louis encephalitis virus antibody, IgM or IgG; anti-lymphocytic choriomeningitis virus antibody, IgM or IgG

**Table S4. Feature selection data for immune variables for logistic regression model predicting atypical vs. classical ME/CFS subgroup membership.***

| **Immune**  **molecule** | **Lasso** | **Random forests** | | | |
| --- | --- | --- | --- | --- | --- |
|  |  | Mean decrease in accuracy | Rank | Gini index | Rank |
| IL1ra | 0 | 0.0003 | 32 | 0.2892 | 24 |
| IL1α | 0 | -0.0006 | 51 | 0.2067 | 36 |
| IL1β | 0 | 0.0003 | 33 | 0.1924 | 40 |
| IL2 | 0 | 0.0000 | 39 | 0.1140 | 49 |
| IL4 | 0 | 0.0006 | 27 | 0.2485 | 31 |
| IL5 | 0 | 0.0030 | 10 | 0.3969 | 13 |
| IL6 | 0 | 0.0045 | 6 | 0.4575 | 9 |
| IL7 | -1.5140 | 0.0183 | **2** | 1.3042 | **3** |
| CXCL8 (IL8) | 0 | 0.0007 | 26 | 0.3808 | 15 |
| IL10 | 0 | 0.0008 | 23 | 0.2485 | 30 |
| IL12p40 | 0 | 0.0012 | 16 | 0.1741 | 43 |
| IL12p70 | 0 | 0.0000 | 38 | 0.1802 | 42 |
| IL13 | 0 | 0.0054 | 5 | 0.6030 | 6 |
| IL15 | 0 | 0.0001 | 36 | 0.1585 | 45 |
| IL17A | -2.3583 | 0.0150 | **3** | 1.3188 | **2** |
| IL17F | 0 | 0.0001 | 35 | 0.1011 | 50 |
| IFNα2 | 0 | 0.0028 | 11 | 0.3591 | 17 |
| IFNβ | 0 | -0.0003 | 47 | 0.1593 | 44 |
| IFNγ | 0 | 0.0014 | 15 | 0.2159 | 34 |
| TNFα | 0 | 0.0009 | 21 | 0.2718 | 25 |
| TNFβ | 0 | -0.0002 | 42 | 0.2495 | 29 |
| CD40L | 0 | 0.0006 | 29 | 0.2705 | 26 |
| sFasL | 0 | 0.0008 | 24 | 0.3502 | 18 |
| TRAIL (TNFSF10) | 0 | 0.0011 | 18 | 0.4259 | 11 |
| CCL2 (MCP1) | 0 | 0.0000 | 37 | 0.2515 | 28 |
| CCL3 (MIP1α) | 0 | 0.0010 | 20 | 0.3875 | 14 |
| CCL4 (MIP1β) | 0 | -0.0006 | 50 | 0.2015 | 39 |
| CCL5 (RANTES) | 0 | 0.0001 | 34 | 0.3057 | 23 |
| CCL7 (MCP3) | 0 | 0.0018 | 14 | 0.4491 | 10 |
| CCL11 (eotaxin) | 0 | 0.0018 | 13 | 0.3352 | 19 |
| CXCL1 (GROα) | 0 | -0.0002 | 41 | 0.2631 | 27 |
| CXCL5 (ENA78) | 0 | -0.0003 | 43 | 0.0796 | 51 |
| CXCL9 (MIG) | 0 | 0.0299 | **1** | 2.1017 | **1** |
| CXCL10 (IP10) | 0 | -0.0003 | 44 | 0.1870 | 41 |
| TGFα | 0 | -0.0005 | 49 | 0.2036 | 37 |
| TGFβ | 0 | 0.0009 | 22 | 0.3240 | 21 |
| SCF (SF) | 0 | 0.0021 | 12 | 0.4097 | 12 |
| CSF1 (MCSF) | 0 | 0.0012 | 17 | 0.3660 | 16 |
| CSF2 (GMCSF) | 0 | 0.0004 | 31 | 0.2328 | 33 |
| CSF3 (GCSF) | 0 | 0.0038 | 7 | 0.4615 | 8 |
| PDGFBB | 0 | -0.0004 | 48 | 0.1432 | 48 |
| bNGF | 0 | 0.0033 | 8 | 0.7910 | 4 |
| FGFb | 0 | 0.0031 | 9 | 0.7111 | 5 |
| HGF | 0 | -0.0003 | 45 | 0.1522 | 47 |
| VEGFA | 0 | -0.0003 | 46 | 0.1528 | 46 |
| LIF | 0 | -0.0001 | 40 | 0.2370 | 32 |
| Resistin | 0 | 0.0008 | 25 | 0.3270 | 20 |
| Leptin | 0 | 0.0005 | 30 | 0.2021 | 38 |
| Serpin E1 (PAI1) | 0 | 0.0087 | 4 | 0.5160 | 7 |
| sICAM1 (CD54) | 0 | 0.0006 | 28 | 0.2145 | 35 |
| VCAM1 (CD106) | 0 | 0.0010 | 19 | 0.3207 | 22 |

**Key:** **ME/CFS**, myalgic encephalomyelitis/chronic fatigue syndrome

* Atypical ME/CFS, n=19 (excludes 8 subjects later developing cancer); Classical ME/CFS, n=32

**Figure S1. Graphic comparison of CSF levels of cytokines in atypical ME/CFS cases, including all exposure/comorbidity categories (cancer [n=8], probable infection [n=5], immune/inflammatory [n=7], other [n=7]), and classical ME/CFS cases (n=32).**


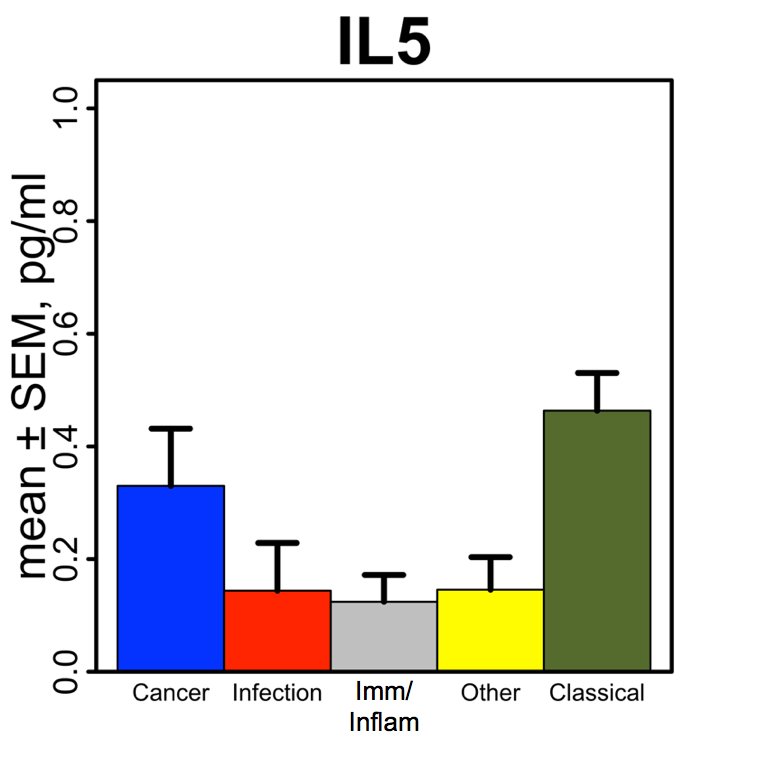

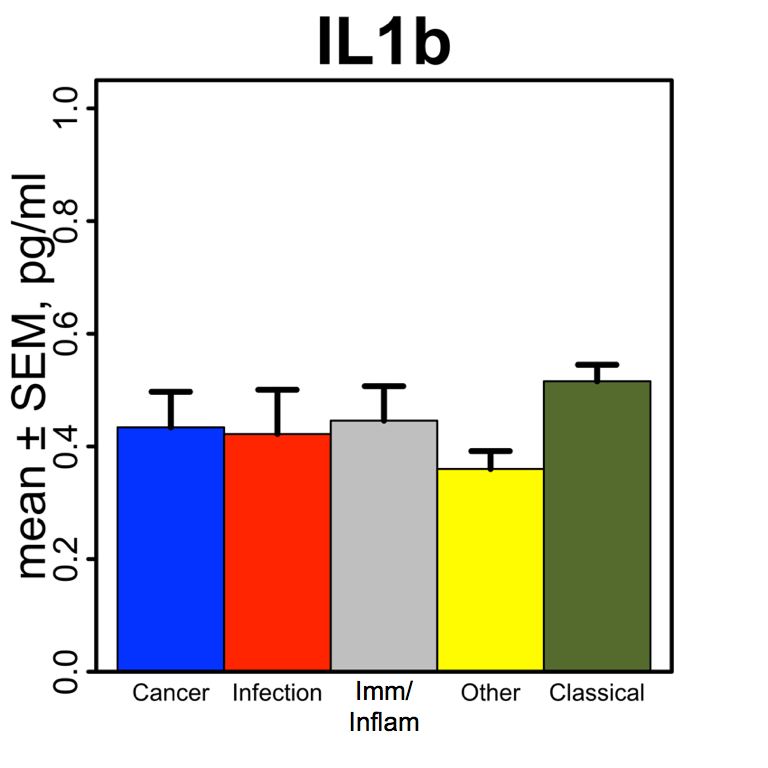

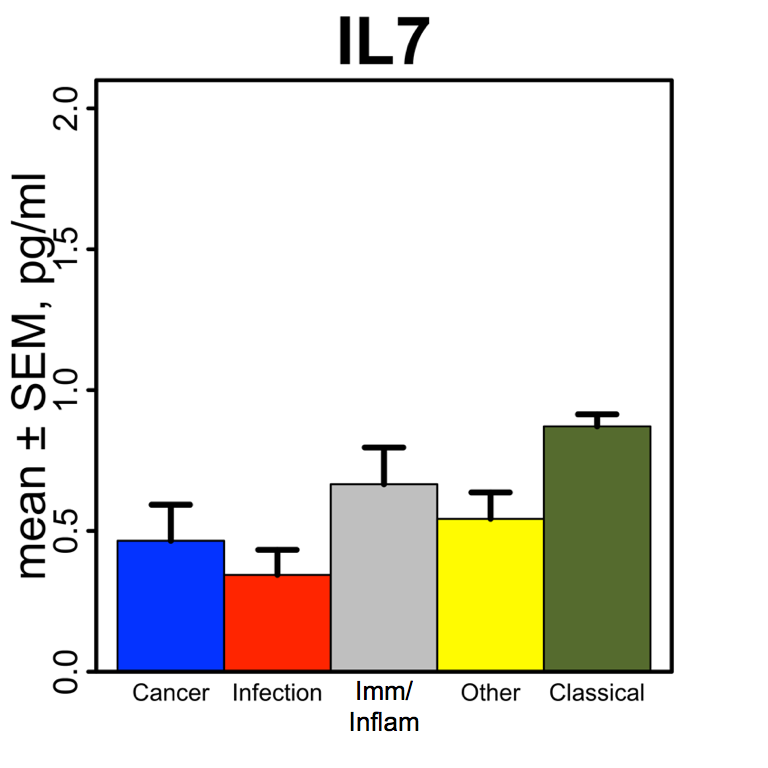

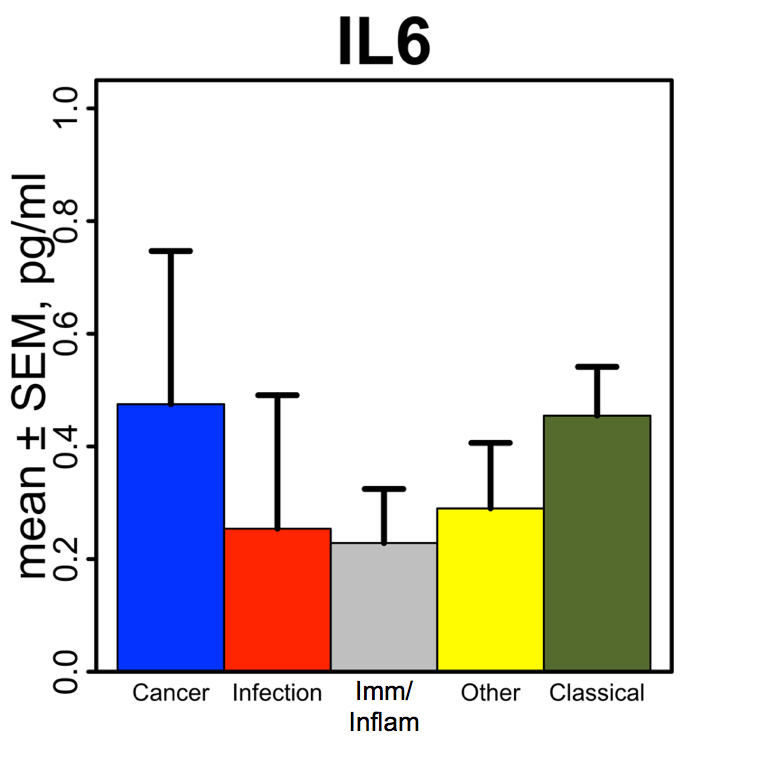

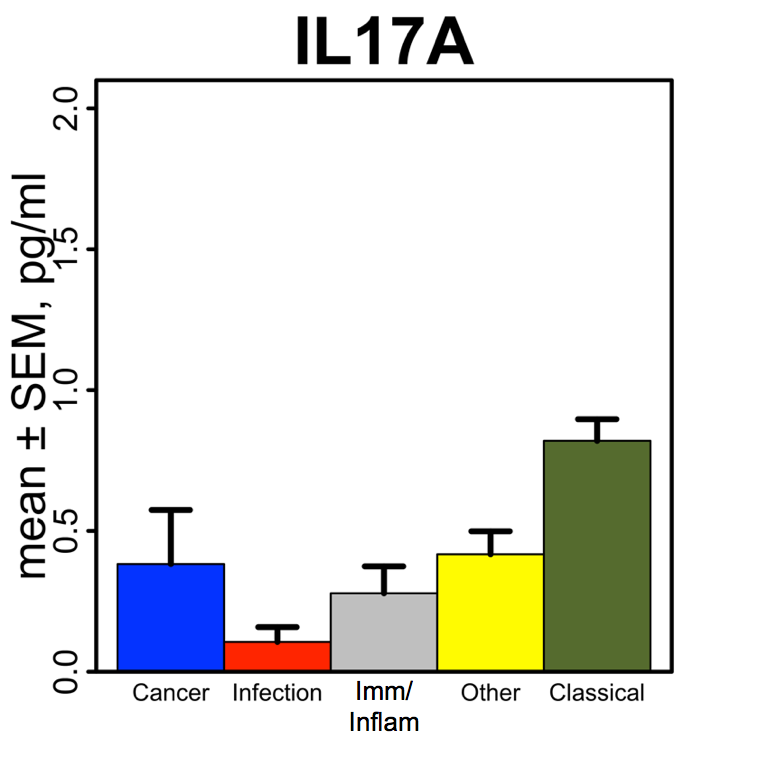

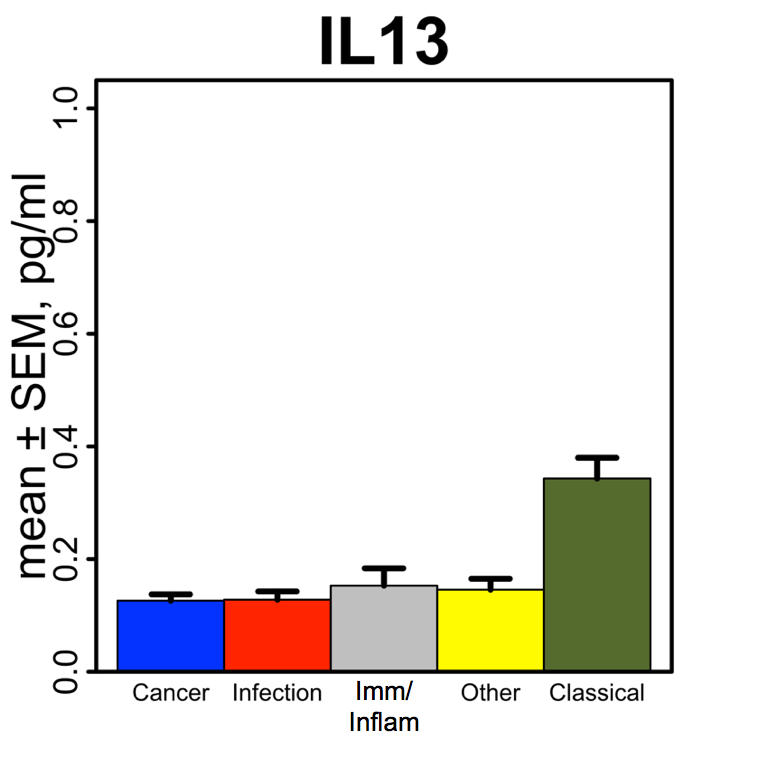

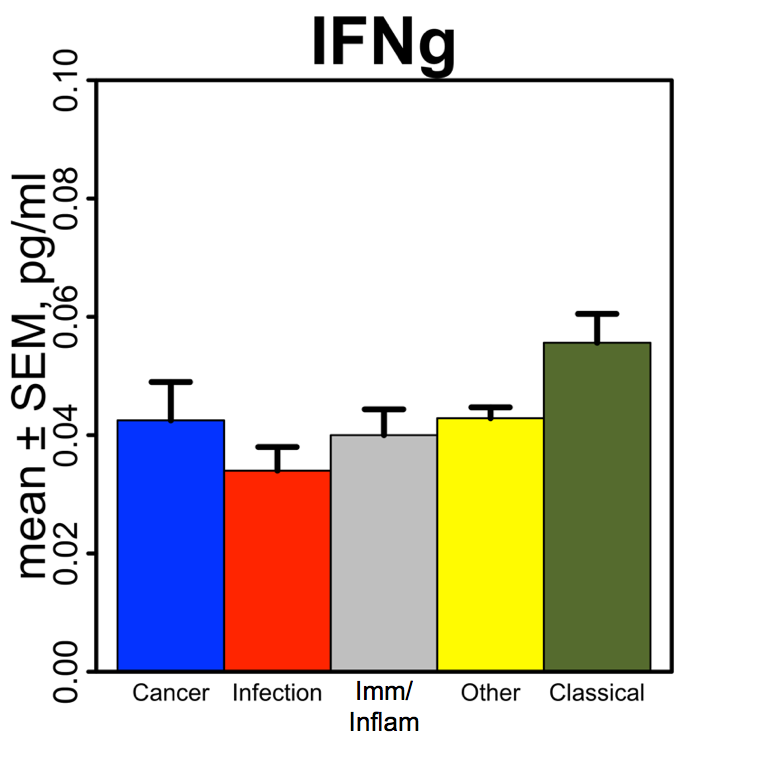

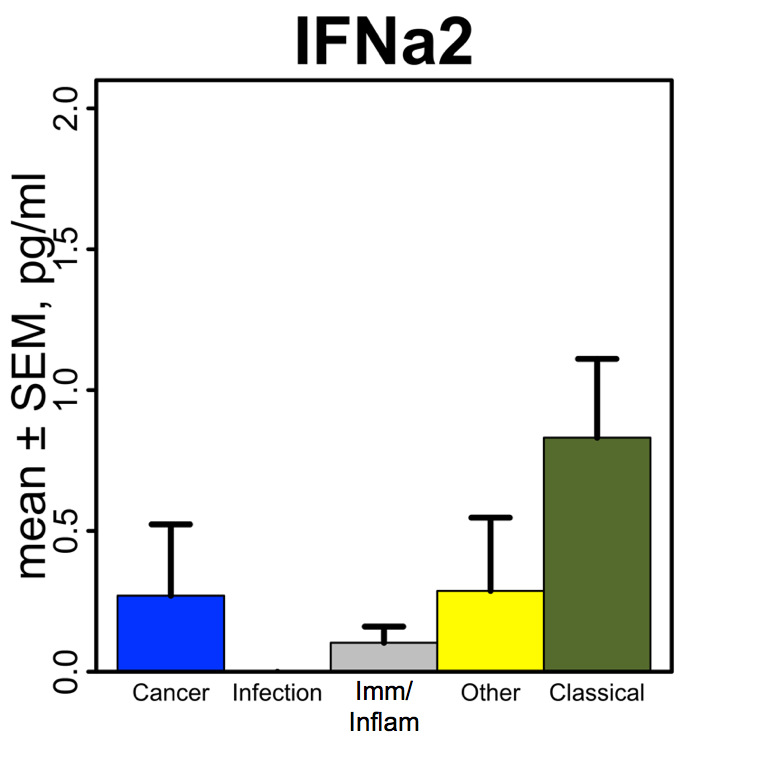

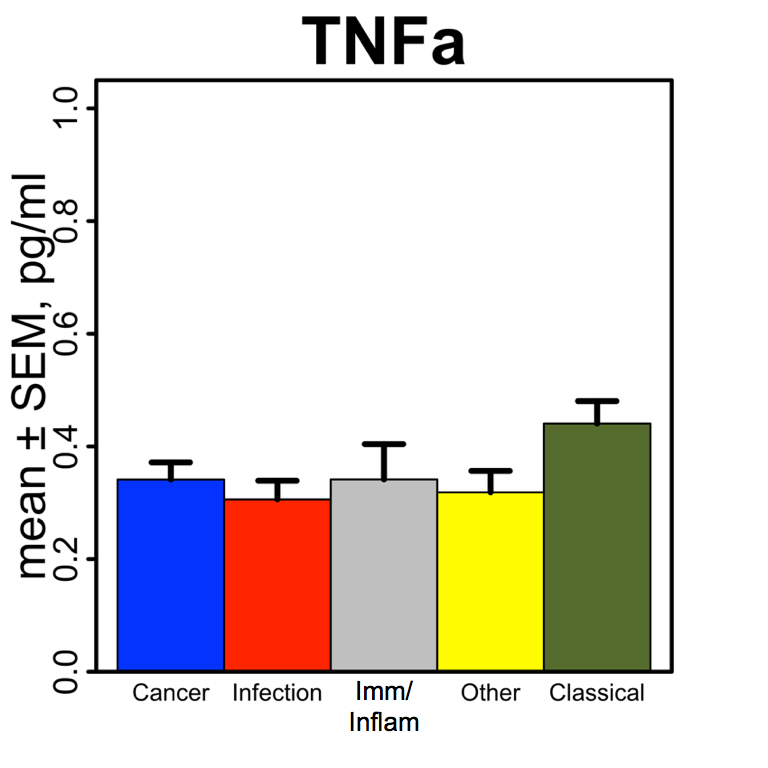

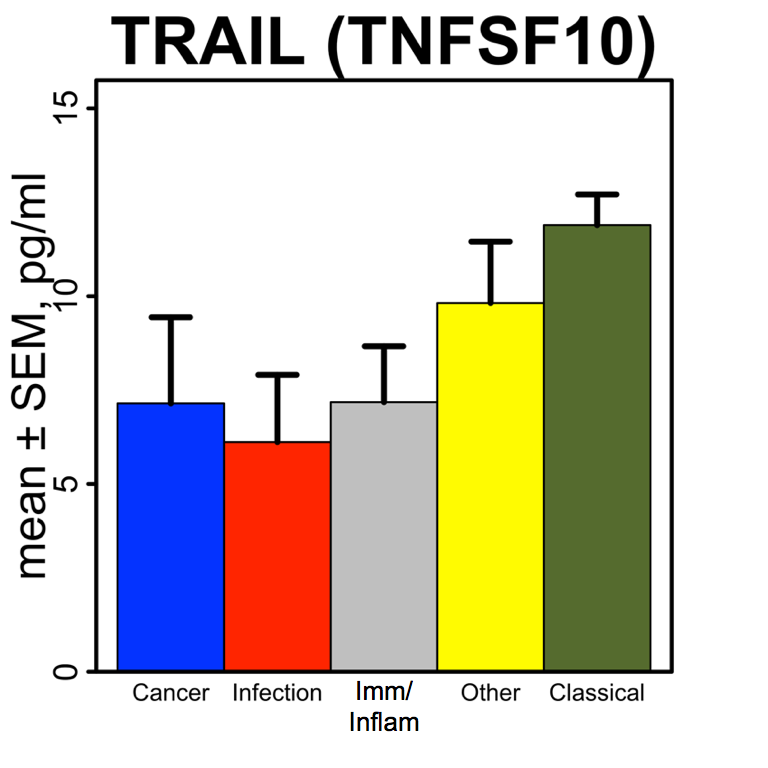

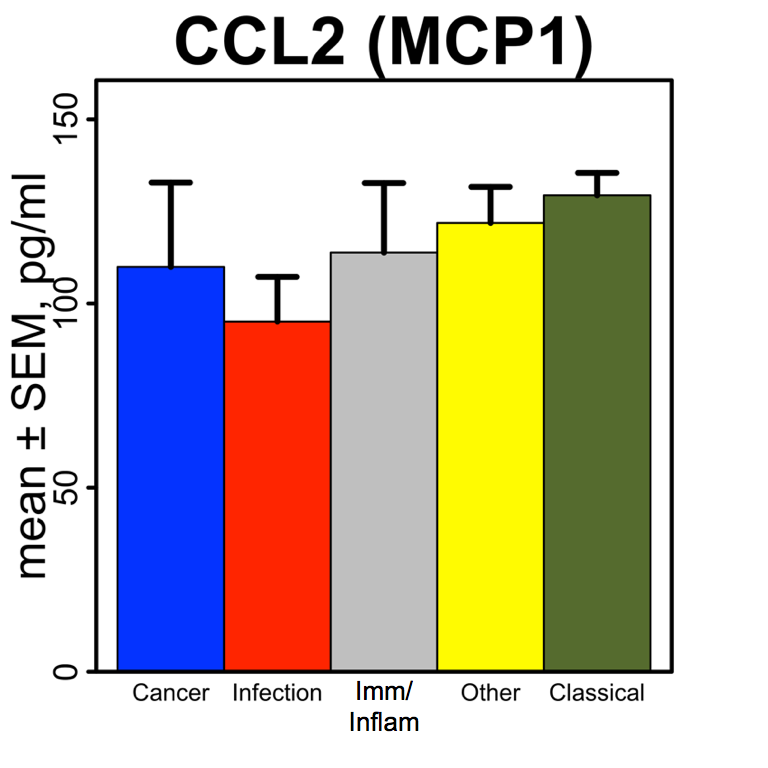

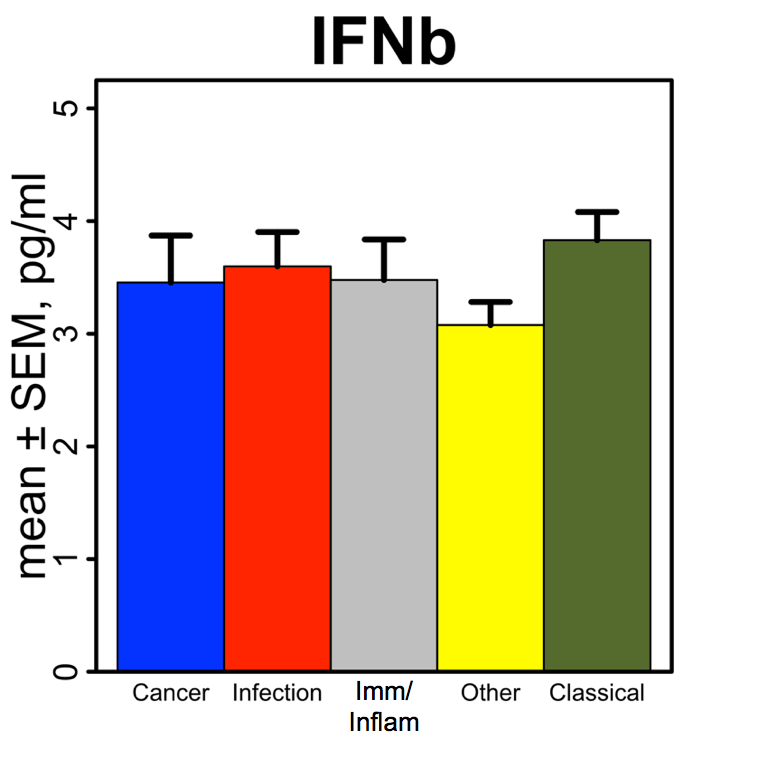


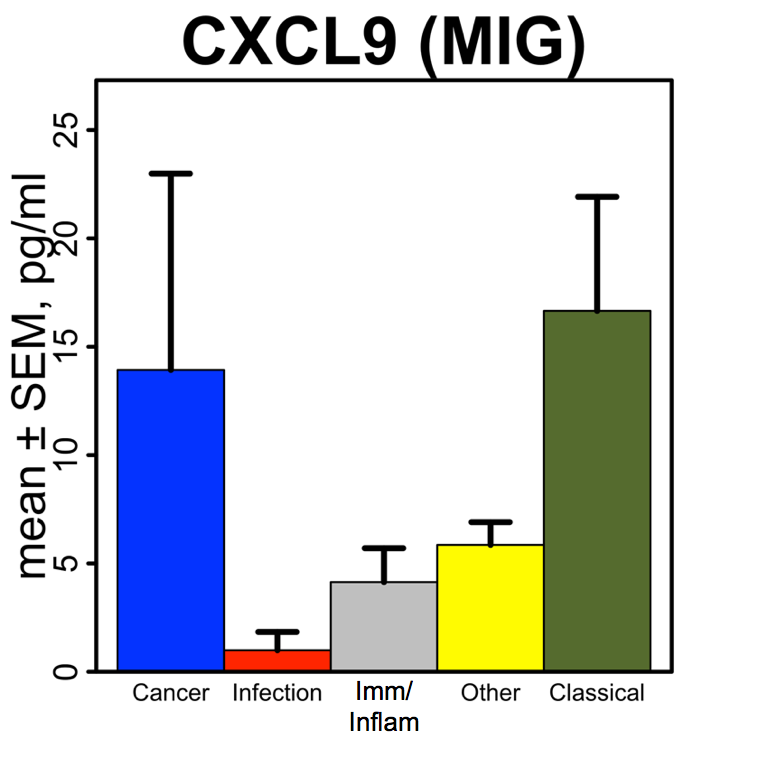

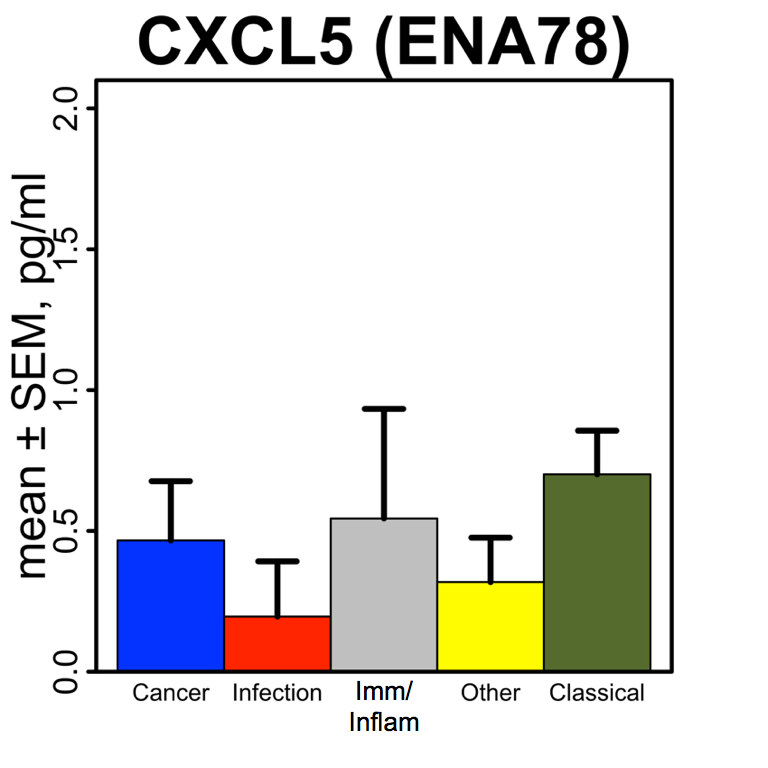

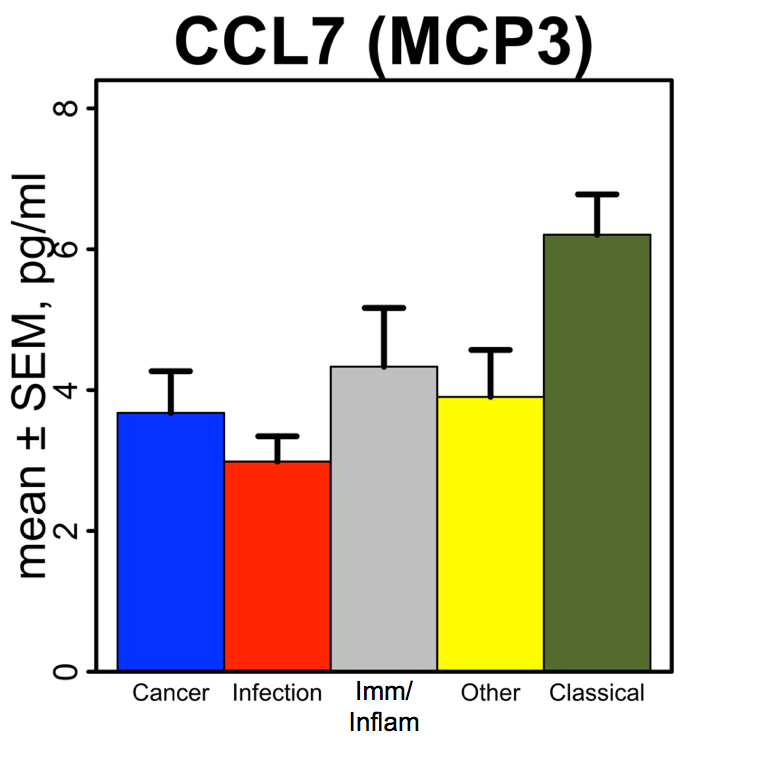

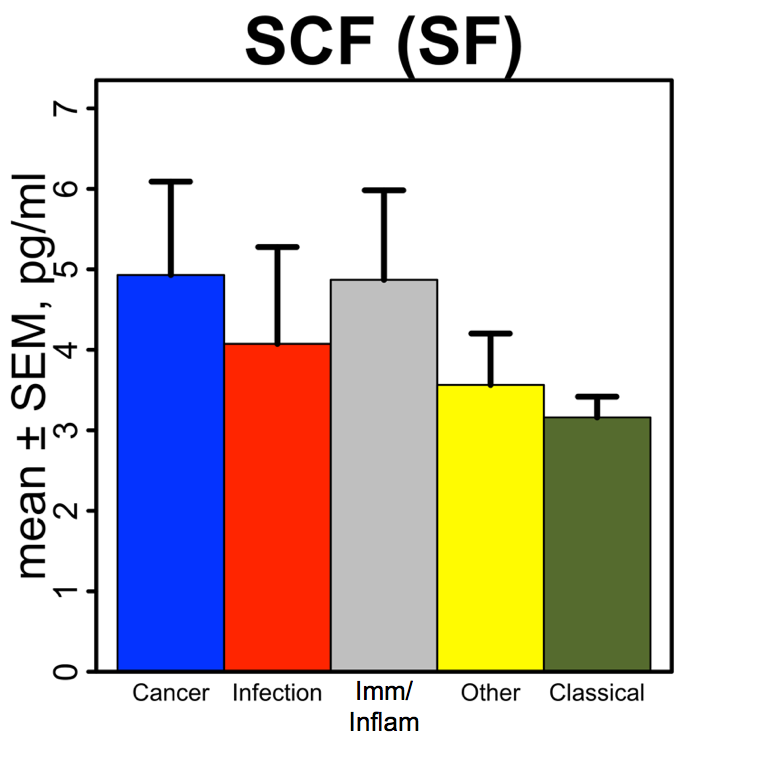

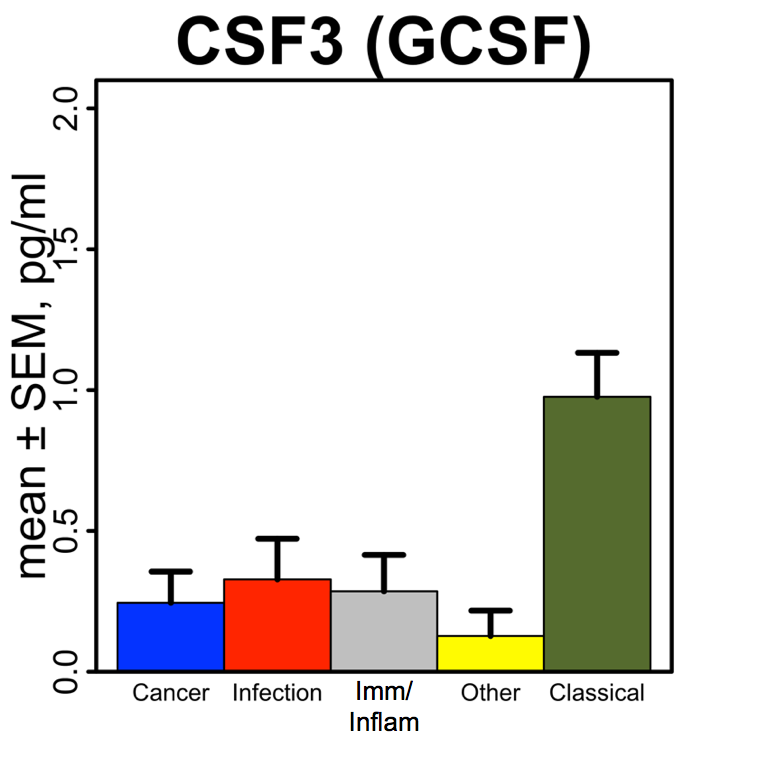

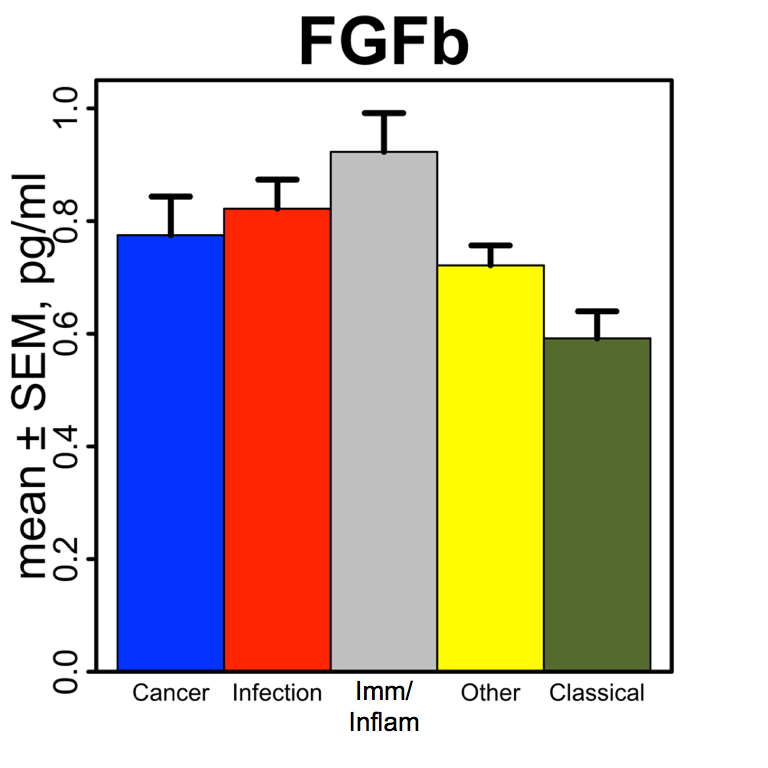

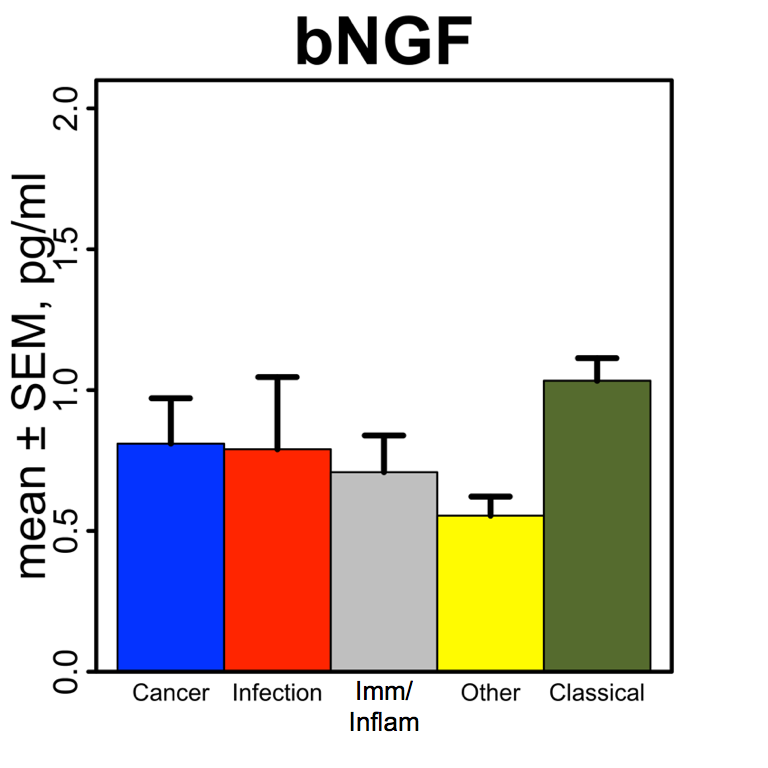

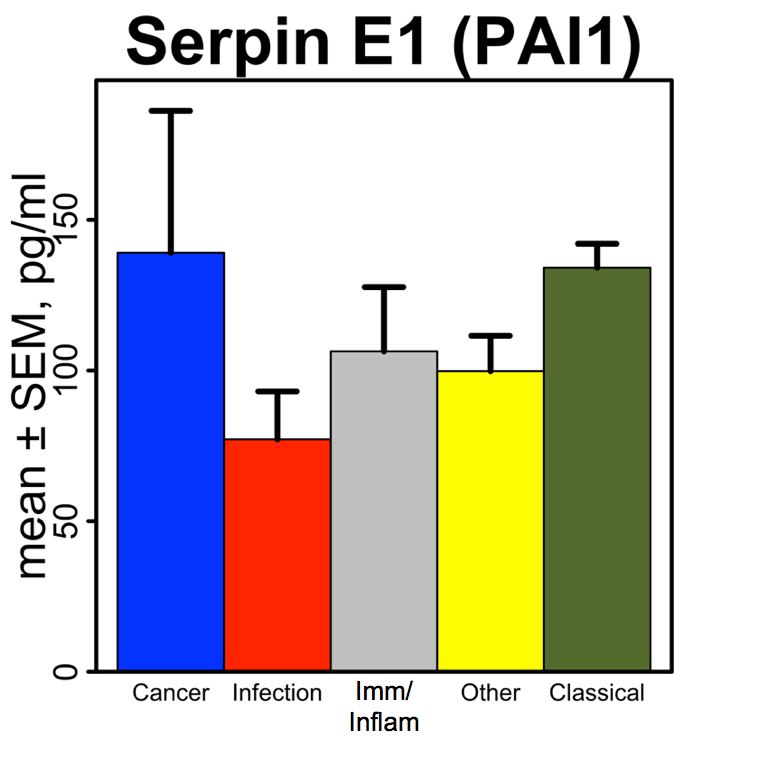

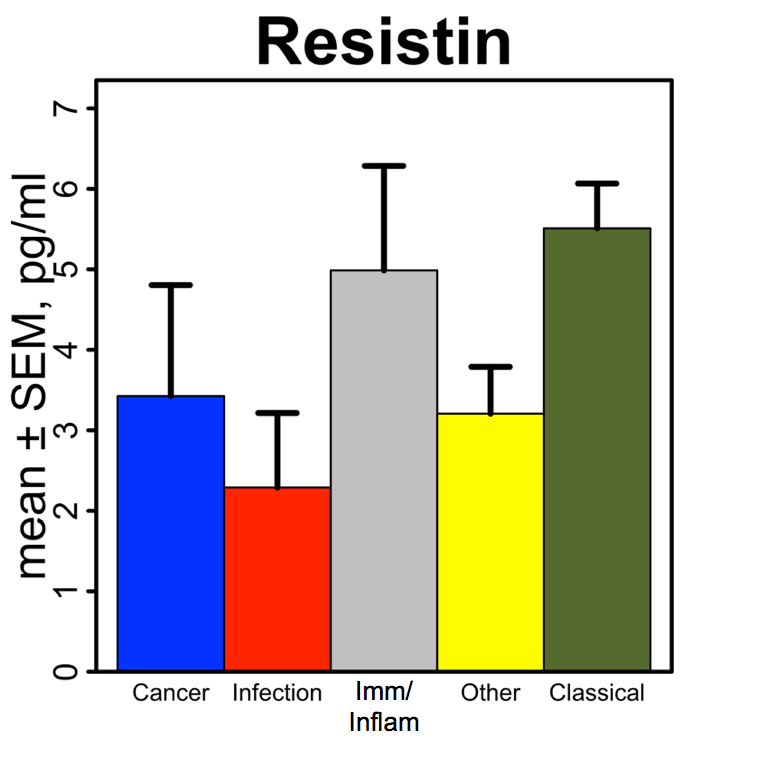


**References**

Cohen J. Statistical power analysis for the behavioral sciences. 2nd ed. Hillsdale, N.J.: L. Erlbaum Associates; 1988.
